# Psychological help-seeking behaviours amongst those living with Inflammatory Bowel Disease; A cross-sectional, descriptive, correlational study

**DOI:** 10.1371/journal.pone.0346243

**Published:** 2026-04-10

**Authors:** Clodagh Byron, Nicola Cornally, Aileen Burton, Elaine Lehane

**Affiliations:** 1 Department of Gastroenterology and Hepatology, Cork University Hospital, Wilton, Cork, Republic of Ireland; 2 School of Nursing and Midwifery, University College Cork, Brookfield Health Sciences Complex, Cork, Republic of Ireland; University of Diyala College of Medicine, IRAQ

## Abstract

**Aim:**

To explore the help-seeking preferences and behaviours of those living with Inflammatory Bowel Disease for negative emotions.

**Design:**

A cross-sectional, descriptive, correlational study.

**Methods:**

The sample consisted of 376 individuals living with Inflammatory Bowel Disease. Quantitative and qualitative data were collected via a questionnaire comprising both researcher-developed items and a validated patient-reported outcome measure. Descriptive and inferential statistics, including correlational and regression analysis were used for quantitative data. Qualitative latent content and count analysis was carried out on the textual data from open-ended survey items.

**Results:**

Participants reported moderately high intentions to seek help for negative emotions related to their Inflammatory Bowel Disease (M = 4.86, SD = 1.80), yet 59.8% had not done so despite experience such emotions. Prior help-seeking was reported by 45%, and 18.6% were currently receiving support. Regression analysis showed that attitudes, subjective norms, and perceived behavioural control explained 55.5% of the variance in behavioural intention, with subjective norms and attitudes as significant predictors. Previous help-seeking experience and surgical history were linked to greater intention to seek help, in addition to diagnosis, with individuals living with Crohn’s Disease reported significantly higher help-seeking intentions than those with Ulcerative Colitis. Common barriers included lack of service awareness, wanting to solve the problem independently, and stigma. Participants strongly value accessible, affordable, and Inflammatory Bowel Disease-informed psychological support, integrated within routine Inflammatory Bowel Disease care and complemented by peer and community resources.

**Conclusion:**

The findings underscore the need for routine assessment of the psychological impact of living with Inflammatory Bowel Disease, timely referral to mental health services, and the integration of psychological care within standard Inflammatory Bowel Disease pathways. Implementing these changes is essential to delivering equitable, patient-centred care. The findings provide behavioural insight into factors associated with seeking help for negative emotions related to Inflammatory Bowel Disease and add to the growing body of literature emphasising the relevance of viewing psychological wellbeing as an important component of Inflammatory Bowel Disease care.

## Introduction

Inflammatory Bowel Disease (IBD) can have a significant negative impact on the psychological wellbeing of those living with the disease [1]. Living with IBD presents multiple challenges, including physical symptoms such as diarrhoea, abdominal pain, incontinence and rectal bleeding, alongside psychological and social challenges that affect relationships, intimacy, and social participation [2]. A qualitative study involving five focus groups and 17 individuals with IBD further highlighted a general societal lack of awareness about IBD and its personal impact, as well as significant psychosocial consequences such as fear of incontinence and subsequent embarrassment, which limited participants’ ability to travel or attend social events and led to feeling of social isolation [3]. The financial burden of IBD also emerged as a prominent finding. Participants reporting difficulties maintaining full-time employment, having to retire early and financial strain due to disease-related costs. These challenges had a negative impact on family dynamics and placed pressure on relationships. Additionally, frustration was expressed at healthcare professionals’ limited understanding of the condition [3]. A secondary aim of the meta-synthesis [2] was to identify strategies used by those living with IBD to manage the challenges faced. However, data on such strategies were sparse. There was also little evidence that people with IBD actively sought help from healthcare professionals to support self-management of challenges experienced outside of scheduled care. An exploration of the help-seeking experiences of patients living with IBD for the challenges associated with their disease from healthcare professionals (n = 8) identified that the physical symptoms associated with IBD impacted negatively on their psychological wellbeing and there was an unmet need for psychological support [4].

IBD may have a profound negative impact on mental health [5], with many experiencing negative emotions, defined as “unpleasant, often disruptive, emotional reaction designed to express a negative affect” [6]. Characteristically, experiencing negative emotions are not conducive in achieving personal objectives [6]. Examples of negative emotions include sadness, anxiety, guilt, loneliness, disappointment, fear, self-criticism, rejection, jealousy, insecurity, uncertainty, or anger. The term ‘negative emotions’ was intentionally used to capture subclinical psychological distress that may not meet the criteria for formal psychiatric diagnoses but nonetheless influences wellbeing and help-seeking behaviour. The term was chosen to explore help-seeking intentions and behaviours at a point that is more closely aligned with the everyday patient experience, but also where formal pathways do not currently exist that might for conditions such as clinically diagnosed depression or anxiety. In relation to previous research, a non-interventional study using self-administered questionnaires was conducted [7] to assess multiple psychosocial factors including satisfaction with life, coping mechanisms, negative emotions and mental recognition of the disease, amongst others. Those diagnosed with IBD (n = 30), experienced more negative emotions and had higher levels of functional negative emotions when compared to healthy controls (n = 30). Fatigue and arthralgia resulted in higher levels of negative emotions. Patients diagnosed with Crohn’s Disease (CD) experienced the least positive emotions. Those living with IBD experienced more anxiety than healthy counterparts, highest amongst those living with Ulcerative Colitis (UC). Greater sadness was also experienced by those living with IBD when compared to the control group [7]. Self-conscious emotions in fifteen individuals living with IBD were explored using semi-structured interviews [8]. Participants reported that a loss of control or visible symptoms threatened their preferred identity, triggering fear, anxiety, shame and guilt, especially when the disease negatively affected others. The authors highlighted the need for further research to focus on the shaping of psychological and therapeutic supports for those living with IBD [8].

While higher rates of psychiatric distress [9], depression, anxiety [10], bipolar disorder [11], schizophrenia [12], and a trend towards increased mortality caused by suicide [13, 14] are observed in the IBD population, less attention has been given to how those living with IBD respond to the earlier negative emotions which may not necessarily meet diagnostic thresholds. Understanding help-seeking at this stage may be particularly important, as left unaddressed, these emotions, although not clinical disorders in themselves, may accumulate and contribute to significant distress or even progress to psychiatric illness over time.

Despite growing recognition of the psychological impact of IBD, access to psychological support for individuals with IBD remains inconsistent internationally, with many relying on peer support through charities such as ‘Crohn’s and Colitis Ireland’ (CCI), ‘Crohn’s and Colitis UK’ and ‘Crohn’s and Colitis Canada’. In relation to clinical guidelines, the European Crohn’s and Colitis Organisation recommend routine assessment of psychosocial impact and quality of life at clinic visits, with referral to psychotherapy and integrated psychosomatic care where needed, and signposting of patient support groups [15]. Similarly, the British Society of Gastroenterology advocate offering psychological therapies to interested patients, particularly those demonstrating psychological symptoms, and that a psychologist form part of the wider multidisciplinary team (MDT) [16]. The American Gastroenterological Association also recommend routine assessment of health-related quality of life, symptom-specific anxiety, early life adversity and functional impairment [17] and that mental health providers form part of the MDT [18]. However, the 2024 IBD patient survey in the United Kingdom found only 20% of patients were asked about mental health, and just 6% of the 117 services that participated had psychologist input at 0.5 whole-time equivalent [19]. A large Australian study reported that while 50% of participants living with IBD experienced psychological distress, only 15.2% were currently receiving care from a mental health practitioner [20]. While 41% of the total participants (n = 108) of older adults living with cardiovascular disease, respiratory disease and/or type 2 diabetes did not intend to see help for mental health [21]. Several barriers have been identified that prevent individuals with IBD from accessing or utilising psychological support. Participants (n = 187) of a survey most frequently cited time constraints (28%), lack of knowledge regarding service availability (25%), and cost (19%) as barriers to accessing psychotherapy [22]. Less frequently reported were stigma (14%), lack of motivation (12%), and emotional concerns (7%) [22].

Although the prevalence of psychological distress and service gaps have been well documented in the literature, far less is known about the behavioural processes that contribute to help-seeking for negative emotions related to IBD. Existing studies have largely focused on prevalence, service availability, or reported barriers, rather than examining how those living with IBD decide whether or not to seek help for negative emotions. In particular, help-seeking intentions for negative emotions related to IBD, the psychosocial factors that influence these intentions, and the relationship between intention and past help-seeking experiences remain underexplored. Furthermore, little research has applied behavioural theory to understand help-seeking for subclinical psychological distress that may not meet diagnostic thresholds but nevertheless impacts wellbeing.

To address this gap, the present study is guided by the theory of planned behaviour (TPB). The TPB was developed by Icek Ajzen [23] as an extension of the Theory of Reasoned Action, incorporating perceived behavioral control to account for behaviours not entirely under volitional control [24]. Typically, the greater the intention to perform the behaviour, the more likely its performance. The intention is governed by three independent concepts, namely attitudes, subjective norms and perceived behavioural control. The attitude towards the behaviour may be defined as the person’s appraisal of the behaviour. Subjective norm is a social factor which denotes the social pressure the person may be subjected to perform or not to perform the behaviour in question. Perceived behavioural control may be defined as the person’s sensed ability to undertake the behaviour in question, be it easy or difficult. Perceived behavioural control takes into account past experiences in addition to expected obstacles. In essence, a positive attitude, favourable subjective norms and perceived behavioural control result in intention to undertake the behaviour in question [24]. The TPB has previously been applied to help-seeking research for both mental health [25] and IBD [26], supporting its applicability in this context and providing the rationale for its selection as the guiding theoretical framework. Grounding this inquiry in theory is imperative as it provides conceptual and methodological rigour and supports the accumulation of knowledge across studies [27].

### The Study

#### Aim.

To explore the help-seeking preferences and behaviours of those living with IBD for negative emotions, within six months of experiencing such emotions.

### Objectives

The objectives of this study were as follows:

1. To evaluate to what extent the constructs proposed by the TPB predict and explain participants’ intentions to seek psychological help for negative emotions.
2. To describe barriers to accessing psychological help for negative emotions related to IBD.
3. To explore preferences for how current services could better support help-seeking for negative emotions related to IBD.

## Methods

### Design

This study employed a cross-sectional, descriptive, correlational design with a quantitative-dominant mixed methods instrument, incorporating an embedded qualitative component to complement the quantitative findings. The Strengthening the Reporting of Observational Studies in Epidemiology (STROBE) guideline for cross-sectional studies guided the reporting [28].

### Study setting and sampling

A minimum sample size of 381 participants was calculated (OpenEpi® version 3.01) based on an estimated population size of 40,000 people living with IBD in Ireland, a confidence level of 95% and a margin of error of 5% [29]. Green’s recommendations [30] were utilised to assess adequacy for the multiple regression analysis (N ≥ 50 + 8m to test the overall model, N ≥ 104 + m for testing individual predictors). In the hierarchical regression model, 15 predictors (m) were included. Therefore, the recommended minimum sample size was 170 for testing the overall model and 119 participants for testing individual predictors. A total of 280 participants were included in the regression indicating that the study was adequately powered to conduct the regression analysis.

### Inclusion and/or exclusion criteria

Inclusion criteria required participants to be aged 18 or over, have a diagnosis of IBD (UC, CD or IBD-Unclassified), and reside within the Republic of Ireland or Northern Ireland. Exclusion criteria included individuals under 18 years of age, those diagnosed with conditions other than IBD such as Irritable Bowel Syndrome, diverticular disease, colon cancer etc., or those residing outside the island of Ireland.

### Data collection

Data were collected using an online questionnaire that contained researcher-developed items in addition to a validated patient reported outcome measure (IBD-Control-8) between the 19^th^ of December 2024 and the 13^th^ of February, 2025 via the online platform Qualtrics©. The mixed-method instrument contained seven sections. Section one screened for eligibility; section two collected clinical characteristics. Section three measured self-perceived disease control using ‘IBD-Control-8’ [31] where scores of ≥13 indicated quiescent disease. The instrument has demonstrated good reliability (Cronbach’s alpha 0.84–0.86 [32, 31]) and validity across languages [33–35]. Section four addressed previous or current sources of support to deal with negative emotions, barriers to seeking support, and informal sources of support, derived from previous literature [2–4, 22]. Section five contained 11 items (across 4 subscales) assessing TPB constructs using established guidance [36]. Direct attitudes were assessed using a seven-point Likert scale comprising four bipolar evaluative items, including instrumental, experiential, and overall evaluation measures. Subjective norms were measured using three items referring to the opinions of important people. Perceived behaviour control was measured using a three-item scale (two self-efficacy, one controllability) and behavioural intention was assessed using a three-item scale. Items were arranged so that the ends of the scales were both a mix of positive and negative endpoints [36]. Section six collected qualitative data concurrently and included one open-ended question inviting participants to express their preferences for how current services could better support help-seeking for negative emotions related to IBD. Section seven gathered socio-demographic data, mental health diagnoses and co-morbidities.

Internal consistency for the TPB scales was assessed using Cronbach’s alpha. The four-item attitude (⍺ = 0.75) and three-item behavioural intention (⍺ = 0.90) scales showed acceptable to excellent reliability [37], with inter-item correlations ranging from 0.31–0.65 and 0.68–0.89, respectively. The three-item subjective norm scale had lower internal consistency (⍺ = 0.58), removing one item (section 5, question 14) increased alpha only marginally (by 0.02), so the item was retained. Inter-item correlations ranged from 0.25–0.43. Perceived behavioural control initially showed low reliability (⍺ = 0.51) [38] but improved to 0.71 after removing one controllability item (section 5, question 7), with moderate inter-item correlations (0.37–0.50).

The online questionnaire was advertised on social media platforms such as Instagram, Facebook and TikTok via ‘CCI’ and on patient-led support groups. The patient-led support groups were selected based on their locations. Only groups in Ireland were requested to advertise the questionnaire to meet inclusion criteria. Moderators of the social media groups were requested to re-advertise the questionnaire every fourteen days. Strategies utilised to optimise online engagement and increase response rates included using a simple design on Qualtrics©, providing an accurate estimation of completion time [39], an accurate progress indicator, and ensuring accessibility using a smartphone, tablet, or computer. More complex designs may require higher-speed internet connections [40], and therefore, a simple design was chosen. A dark text over a white background was applied as this is thought to increase response rates due to the similarity to pencil and paper [41]. Participation was voluntary and no incentives were offered.

### Methodological rigor and bias control

The questionnaire underwent content validity and reliability assessment prior to deployment online, following established guidelines [42]. An expert panel composed of a Clinical Nurse Specialist (n = 1), a candidate Advanced Nurse Practitioner (n = 1), an Advanced Nurse Practitioner (n = 1), an IBD Research Nurse (n = 1), Academic Nurse Researchers (n = 2), Consultant Gastroenterologists (n = 3) and lay representatives with IBD (n = 2) assessed item necessity (Content Validity Ratio), relevancy and clarity (Content Validity Index), including both item-level (I-CVI) and scale-level (S-CVI-Ave), leading to revisions and the elimination of the ‘Barriers to Access Care Evaluation’ scale. The revised questionnaire was re-reviewed by the expert panel prior to dissemination.

Participants were recruited using a non-probability, voluntary response sampling method, which may have introduced selection, sampling, and non-response bias [43]. Although the questionnaire was advertised across multiple social media platforms to diversify the population reach [44], this strategy does not eliminate the risk of bias. Similarly, the eligibility criteria was also kept broad to ensure that no particular subgroup of IBD patients was systematically excluded, however representativeness cannot be assumed. The survey was available across devices [45], and participation was fully anonymous to encourage responses [46], though non-response bias cannot be ruled out.

### Ethical considerations

This study obtained ethical approval from Social Research Ethics Committee, University College Cork (reference number 2024−216). All participants provided informed electronic consent before completing the questionnaire. IP addresses, location or identifiable data were not collected by Qualtrics©. The research was conducted in accordance with the Declaration of Helsinki and institutional research guidelines of University College Cork (Code of Research Conduct v.2.4; Guidance Document for Conducting Internet Research).

### Data analysis

## Analysis of variables

Variable selection was theoretically informed by the TPB with the key outcome variable scribed as behavioural intention and the dependent predictor variables attitude, subjective norm and perceived behavioural control. Sociodemographic variables were age, gender identity, assigned sex at birth, education level and access to healthcare provisions. Clinical variables were diagnosis, length of diagnosis, current treatment, surgical history, mental health diagnoses, co-morbidities, perceived disease control (IBD-Control-8), previous help-seeking experiences and barriers to seeking help for negative emotions. Where appropriate, quantifiable variables were grouped for subgroup comparisons, based on clinical indications. Data were summarised using descriptive statistics including frequencies, means, percentages and standard deviations.

### Correlational analysis

To investigate the relationships between attitude, subjective norms, perceived behavioural control and behavioural intention, the spread of data for each scale was assessed using histograms, normal probability plots and formal tests such as Shapiro-Wilks and Kolmogorov-Smirnov. Despite results on the Kolmogorov-Smirnov test suggesting statistically significant deviation from normality (p < 0.001), visual inspection of histograms and Q-Q plots (Figs 1, 2 of S1 File, 6, 7 of S4 File, 8, 9 of S5 File, 10, 11 of S6 File) suggested approximately normal distribution. Skewness values for attitude (−0.40), subjective norms (−0.35), perceived behavioural control (−0.18) and behavioural intention (−0.46) fell within the acceptable range (−0.5 to +0.5). Nevertheless, due to the ordinal nature of the data and the statistical significance of the normality test, non-parametric analyses (Spearman’s rho) were employed [47], increasing the validity of the results [48].

### Comparative analysis

Analyses were then conducted to explore differences in behavioural intention scores across various sociodemographic and clinical variables outlined previously. Due to small sample sizes, certain categories (e.g., “unsure” responses for category of IBD [n = 6], mental health diagnoses [n = 8], and co-morbidities [n = 8]) were excluded from inferential statistics due to potential ambiguity regarding diagnosis. Analyses were limited to larger subgroups where appropriate (e.g., men and women for gender identity) to preserve statistical robustness. However, the exclusion of these categories may limit the generalisability of findings to those uncertain of their diagnostic status or identifying outside binary gender categories. Appropriate statistical tests were selected based on assessment of normality and homogeneity of variance. Where parametric assumptions were met, independent t-tests or ANOVA were used, where assumptions were violated, non-parametric alternatives (Mann-Whitney U and Kruskal-Wallis tests) were employed. Effect sizes were also calculated where relevant to assess the practical significance of the observed differences.

### Multiple regression analysis: standard and hierarchical

Standard multiple regression was conducted to examine the relationship between attitude, subjective norm, and perceived behavioural control on behavioural intention. Hierarchical multiple regression was conducted to examine the contribution of the constructs of the TPB to the prediction of behavioural intention, over and above socio-demographics, clinical characteristics and previous help-seeking experience. One hierarchical multiple regression with four models was constructed to examine predictors of behavioural intention. Model 1 included socio-demographic (age, gender identity, education level, ethnic/cultural background), clinical characteristics (diagnosis, length of diagnosis, current medication(s), previous surgical history, mental health diagnoses, co-morbidities, disease control) and previous experience of seeking help. Models 2–4 added the constructs of the TPB to those variables included in Model 1. Prior to analyses, all assumptions necessary were assessed. This included normality, linearity, multicollinearity, and homoscedasticity. No significant violations of the assumptions of normality or linearity were observed. To strengthen the model and reduce multicollinearity, ‘sex assigned at birth’ was excluded from the hierarchical multiple regression model due to its high collinearity with ‘gender identity’. The removal of this variable enhanced the stability and interpretability of the model, with ‘gender identity’ retained. The assumption of homoscedasticity was met as evidenced by the distribution of residuals.

### Qualitative analysis

Qualitative data was analysed separately to the quantitative data before being integrated at interpretation stage, using latent content analysis [49]. This approach was employed to capture the depth and meaning of participants’ responses [50], and to remain close to the data [51, 52]. The raw data was first divided into meaning units, condensed and coded, before being grouped into categories. These categories were then abstracted into broader themes and subthemes [53]. Coding was conducted by the first author and reviewed by all other authors to enhance credibility and analytical rigour. Emerging codes, categories, and themes were discussed within the research team to ensure consistency and reflexive dialogue, thereby supporting the trustworthiness of the analysis. A count analysis was undertaken to indicate the frequency with which participants referred to subthemes and themes. Each participant could contribute to more than one subtheme, and counts were reported as the number of participants mentioning each subtheme/theme.

### Data handling

Data were exported from Qualtrics© to Statistical Package for Social Sciences (SPSS Mac version 29) prior to representing each variable with a label and code. Recoding, manipulation and computing of new variables was preformed prior to undertaking analysis. Missing data was handled using pairwise deletion for descriptive statistics to maximise the use of all available data [54] and listwise deletion for regression analyses. Sample sizes of each section of the questionnaire varied. For qualitative responses, data management and coding were conducted using Microsoft Excel.

## Results

A total of 437 responses were gathered, of which 376 met eligibility and were retained for analysis. Responses were excluded if participants were ineligible or if eligible respondents did not provide data beyond the initial screening questions.

### Socio-demographic and clinical characteristics of the sample

Participants ranged in age from 18 to 74 years (M = 39.41 years, SD = 11.84). The majority identified as women (62.7%), and 64.4% were assigned female at birth. A substantial proportion of the participants (79.2%) had attained a higher certificate of education or above. The most common diagnosis among participants was CD (n = 208, 55.3%), followed by UC (n = 127, 33.8%). A total of 15.8% (n = 48) reported a co-occurring mental health condition. Additional socio-demographics and clinical characteristics of the sample are presented in Table 1 and 2.

**Table 1:** Socio-demographics of the sample.

Age	% (n)
Young adult (18 –25)	9.9 (30)
Adult (26–44)	59.4 (180)
Middle-age (45 –59)	23.8 (72)
Old age (>60)	6.9 (21)
**Gender Identity**	**% (n)**
Woman	62.7 (190)
Man	35 (106)
Gender queer	1.0 (3)
Nonbinary	0.7 (2)
Agender	0.3 (1)
Gender not specified	0.3 (1)
**Assigned Sex at Birth**	**% (n)**
Female	64.4 (195)
Male	35.6 (108)
**Education Level**	**% (n)**
Lower secondary school	3.3 (10)
Upper secondary school, technical or vocational school	17.5 (53)
Advanced certificate, completed apprenticeship, higher certificate	19.1 (58)
Diploma, ordinary/honours bachelor’s degree	31.4 (95)
Postgraduate diploma/certificate/degree, Doctorate or higher	28.7 (87)
**Healthcare Entitlements**	**% (n)**
Private health insurance	52.6 (159)
Fully subsidised public healthcare (medical card holder)	33.8 (102)
Partially subsidised public healthcare	21.8 (66)
Free GP Care (GP visit card holder)	4 (12)
Subsidised medications and appliances (long-term illness card holder)	2 (6)
**Ethnicity**	**% (n)**
White- Irish	85.8 (260)
White- Any other background	5.9 (18)
Black or Black Irish- African	2.3 (7)
Asian or Asian Irish- Chinese	2 (6)
Black or Black Irish- Any other background	1.3 (4)
Other, including mixed background	1.3 (4)
Asian or Asian Irish- Any other background	0.7 (2)

Socio-demographics of sample. Total n=303, missing data n=73.

**Table 2:** Clinical characteristics of the sample.

Diagnosis^a^	% (n)
Crohn’s Disease	55.3 (208)
Ulcerative Colitis	33.8 (127)
IBD-Unclassified	9.3 (35)
Unsure	1.6 (6)
**Length of Diagnosis** ^**a**^	**% (n)**
<1 year	15.2 (57)
1-2 years	13.8 (52)
3-5 years	22.3 (84)
6-10 years	17.6 (66)
>10 years	31.1 (117)
**Current Treatment** ^**ab**^	**% (n)**
No	15.7 (59)
Yes	84.3 (317)
Infliximab	29.5 (111)
5-ASAs	21.5 (81)
Steroids	12.8 (48)
Adalimumab	12.8 (48)
Immunomodulators	10.9 (41)
Ustekinumab	8.5 (32)
Vedolizumab	7.2 (27)
Small molecules	3.2 (12)
Golimumab	1.9 (7)
Risankizumab	1.3 (5)
**Previous IBD-related Surgical Procedure** ^**bc**^	**%(n)**
No	59.5 (222)
Yes	40.5 (151)
Incision and drainage of fistula	13.3 (50)
Small bowel resection	13.0 (49)
Drain insertion for fistula	11.4 (43)
Right hemicolectomy	7.2 (27)
Panproctocolectomy	6.6 (25)
Temporary stoma formation	4.3 (16)
Total colectomy	3.5 (13)
Ileorectal anastomosis	2.4 (9)
Unsure of surgery type	2.4 (9)
Subtotal colectomy	1.9 (7)
**Previous Mental Health Diagnosis** ^**bde**^	**%(n)**
No	81.5 (247)
Unsure	2.6 (8)
Yes	15.8 (48)
Depression	9 (34)
Anxiety	8 (30)
Post-traumatic stress disorder	1.9 (7)
Eating disorders	1.6 (6)
Other	1.6 (6)
Postpartum mental health conditions	0.8 (3)
Paranoia	0.3 (1)
Steroid induced psychosis	0.3 (1)
Panic disorder	0.3 (1)
Unsure of diagnosis	0.5 (2)
Bipolar disorder	0.3 (1)
Schizophrenia	0.3 (1)
Neurodevelopmental disorders	0.3 (1)
**Co-morbidities** ^**def**^	**% (n)**
No	67.7 (205)
Unsure	2.6 (8)
Yes	29.7 (90)
Diseases of the musculoskeletal system and connective tissues	(19)
Diseases of the circulatory system	(14)
Diseases of the genitourinary system	(13)
Diseases of the skin and subcutaneous tissue	(12)
Diseases of the nervous system	(8)
Malignant neoplasms	(3)
Symptoms, signs and abnormal clinical and laboratory findings, not elsewhere classified	(2)
Disorders of other endocrine glands	(2)
Codes for special purposes	(1)
Diseases of the digestive system	(1)
**Perceived Disease Control (IBD-Control-8)** ^**g**^	**% (n)**
<13	64.3 (232)
≥13	35.7 (129)

Clinical characteristics of the sample.

^a^ (n = 376, missing data n = 0).

^b^ Participants could select multiple options.

^c^ (n=373, missing data n=3).

^d^ (n=303, missing data n=73).

^e^ Not all participants provided a breakdown of diagnosis.

^f^ Qualitative analysis categorised the free text responses of the type(s) of co-morbidities, in accordance with the International Classification of Disease.

^g^ (n=361, missing data n=15).

### Behavioural intention and past help-seeking

Participants demonstrated a moderately high intention to seek help from a healthcare professional for negative emotions related to their IBD within six months of experiencing such emotions (M = 4.86, SD = 1.80). Despite this, a notable proportion of participants (59.8%, n = 201) reported experiencing negative emotions but had not sought help. Of the total sample, 45% (n = 162) has previously sought help, and 18.6% (n = 67) were currently receiving help. Detailed item-level responses and descriptive statistics for behavioural intention are available in Tables 3 and 4 in S1 File. The distribution of formal and informal sources of support is available in Table 5, S2 File.

### Theory of planned behaviour; attitudes, subjective norms, perceived behavioural control and behavioural intention

Overall, participants reported generally positive attitudes towards seeking help from a healthcare professional for IBD-related negative emotions (M = 5.21, SD = 1.23), relatively low level of perceived social pressure (M = 3.87, SD = 1.37), and moderate perceived behavioural control (M = 4.60, SD = 1.40). Statistically significant positive correlations were observed between behavioural intention and attitude (rho = 0.565, p < 0.001), subjective norm (rho = 0.671, p < 0.001), and perceived behavioural control (rho = 0.246, p < 0.001) (n = 306) (Figure 3-5, S3 File). Percentage distribution of responses and additional descriptive statistics for individual items are presented in Tables 6 and 7 in S4 File, 8 and 9 in S5 File, 10 and 11 in S6 File.

### Disease control

Most participants (64.3%, n = 232) did not report quiescent disease at the time of data collection.

### Barriers to accessing help for negative emotions

Participants reported a range of perceived barriers to seeking help for negative emotions related to their IBD. The most frequently endorsed barriers (*agreed* and *strongly agreed*) included a lack of knowledge of available services (67.9%), a preference for wanting to solve the problem independently (63.9%) and feelings of embarrassment or shame (60.2%). Additional commonly reported barriers were difficulties accessing services (57.5%), financial constraints (56.1%), and concerns about not being understood (54.8%). In contrast, fewer participants reported previous negative experiences with mental health services (15.4%), scepticism about the effectiveness of treatment (43.2%), beliefs that other were more deserving of help (44.3%), or challenges attending appointments (47.6%). Supplementary findings on barriers to accessing help are available in Table 12, S7 File.

### Socio-demographic and clinical variables associated with accessing help for negative emotions

Behavioural intention was significantly higher amongst individuals with a history of IBD-related surgery (U = 8247.00, Z = −4.254, p < 0.001), those diagnosed with CD vs. UC (H = 12.615, df = 2, p = 0.002; Bonferroni-corrected alpha = 0.017, p < 0.001), and those with previous experience of seeking help (U = 5648.00, Z = −7.813, p=<0.001). No statistically significant differences were found based on age, gender identity, education level, access to healthcare provisions, length of diagnosis, current treatment, mental health diagnosis, co-morbidities, or perceived disease control.

### Regression

Multiple linear regression indicated that 55.5% of the variance in mean behavioural intention (R² = 0.555, p < 0.001) was explained by the constructs of the TPB. Attitude (B = 0.52, 95% CI [0.38, 0.66], p < 0.001) and subjective norms (B = 0.72, 95% CI [0.61, 0.83], p < 0.001) were significant predictors, whereas perceived behavioural control was not (B = −0.01, 95% CI [−0.12, 0.11], p = 0.904). Subjective norms demonstrated the strongest standardised effect (Beta = 0.547), followed by attitude (Beta = 0.355). Table 3 summarises the multiple regression analysis while Fig 12 (S8 File) displays the associated scatterplot of standardised residuals against standardised predicted values for the standard multiple regression model examining the influence of attitude, subjective norms, and perceived behavioural control on behavioural intention.

**Table 3:** Multiple Regression Analysis.

Model^a^	Unstandardised Coefficients B Std. Error		Standardised Coefficients Beta t		Sig.	95.0% CI Lower Upper	
(Constant)	−0.601	0.336		−1.787	0.075	−1.262	0.061
Mean Attitude	0.520	0.069	0.355	7.553	<0.001	0.384	0.655
Mean Subjective Norm	0.720	0.054	0.547	13.329	<0.001	0.614	0.827
Mean Perceived Behavioural Control	−0.007	0.057	−0.005	−0.121	0.904	−0.120	0.106

^a^Dependent variable: Behavioural intention.

In the hierarchical regression, model 1 (socio-demographics, clinical characteristics and previous help-seeking experience) explained 25.1% of the variance in behavioural intention (R square = 0.251, p < 0.001) with an adjusted R square of 0.217. In this model, only previous help-seeking experience was a significant predictor (p < 0.001). The addition of the constructs of the TPB in subsequent steps significantly improved model fit. Adding attitude in Model 2 increased the explained variance by 15.1% (R square change 0.151), while the inclusion of subjective norm in Model 3 further increased the explained variance by 20.4% (R square change 0.204). The addition of perceived behavioural control in Model 4 contributed only an additional 0.1% (R square change 0.001). The final model (4) explained 60.6% of the variance in behavioural intention (R square = 0.606, p < 0.001, adjusted R square = 0.584), reflecting a 35.5% increase in explained variance (p < 0.001). In this final model, only attitude and subjective norms were statistically significant predictors of behavioural intention (p < 0.001). Table 4 presents the hierarchical multiple regression analysis, while Fig 13 (S8 File) displays the associated scatterplot of standardised residuals plotted against the standardised predicted values for the final model (4) of the hierarchical multiple regression.

**Table 4:** Hierarchical Multiple Regression Analysis.

	Unstandardised B	Coefficients Std. Error	Standardised Coefficients Beta	t	Sig	CI 95% Lower	CI 95% Upper	Collinearity Tolerance	VIF
Constant	1.084	0.884		1.226	0.221	−0.657	2.824		
Length of diagnosis	−0.028	0.060	−0.021	−0.459	0.647	−0.147	0.091	0.683	1.464
Diagnosis	0.050	0.127	0.016	0.391	0.696	−0.201	0.300	0.843	1.187
Current medications	−0.026	0.206	−0.005	−0.128	0.899	−0.431	0.379	0.935	1.070
Previous surgery	−0.006	0.057	−0.005	−0.100	0.920	−0.118	0.107	0.697	1.434
Previous help-seeking experience	−0.658	0.171	−0.178	−3.856	<0.001	−0.993	−0.322	0.698	1.433
Age	0.061	0.115	0.024	0.525	0.600	−0.166	0.287	0.702	1.424
Gender identity	0.026	0.156	0.007	0.165	0.869	−0.282	0.334	0.890	1.123
Education	−0.021	0.063	−0.013	−0.326	0.745	−0.145	0.104	0.947	1.056
Mental health diagnosis	−0.164	0.208	−0.032	−0.788	0.431	−0.573	0.245	0.898	1.113
Ethnic/cultural background	0.034	0.046	0.030	0.741	0.459	−0.057	0.126	0.892	1.121
Other chronic condition(s)	0.033	0.173	0.008	0.193	0.847	−0.307	0.374	0.799	1.251
Mean attitude	0.479	0.074	0.321	6.479	<0.001	0.334	0.625	0.606	1.650
Mean subjective norm	0.687	0.061	0.516	11.319	<0.001	0.568	0.807	0.717	1.396
Mean perceived behavioural control	−0.048	0.063	−0.036	−0.757	0.450	−0.171	0.076	0.656	1.524

### Preferences for psychological care

Analysis of qualitative responses (n = 151), supported by count data, provided rich insights into participants’ suggestions to improve current services in the context of psychological care for IBD. Five themes were identified combining content and frequency-based findings, (1) “enhancing access and affordability of psychological care”, (2) “strengthening IBD service capacity and resources”, (3) “integrating psychological care into IBD care pathways”, (4) “IBD specific expertise in psychological support” and (5) “expanding peer and community-based supports”. Collectively, these themes highlight participants’ desire for psychological care that is accessible, has capacity, embedded in routine care, and tailored to the unique challenges experienced by those living with IBD.

### Enhancing access to and affordability of psychological care

The first theme, reported by 39% participants (n = 59), focused on their ability to access psychological support and contained three subthemes, “cost and affordability” (n = 42, 27.8%), “timely access” (n = 24, 15.9%), and “convenience and flexibility” (n = 5, 3.3%). Participants highlighted the “prohibitive” (#132) cost of psychological care, reporting that they had to “sacrifice other pursuits to pay for it” (#148). Others emphasised the importance of “free access” (#007) to services when needed. Timely access was also a significant concern, as long waiting lists for public services left participants without support or forced to pay privately, “I am paying privately as healthcare waiting lists are long. When I asked my nurse she said I could look into it but would be a while” (#071). Convenience and flexibility in the delivery of services were also prioritised, with participants calling for appointments “at a time that would fit around work commitments” (#053) and access to online supports for those “working or who find it financially difficult attending appointments in person” (#121).

### Strengthening IBD service capacity and resources

The second theme focused on healthcare system capacity (n = 28, 18.5%), specifically within the IBD team and contained two subthemes, “increased IBD nurse capacity” (n = 23, 15.2%) and “longer consultation times” (n = 5, 3.3%). Many participants stressed the need for “more specialist nurses” (#110), describing them as “helpful” (#123), “easy to talk to” (#143), yet overstretched, “so many other parts to their role” (#088). Others identified the need for longer consultation times, requesting more time with their IBD team to discuss not only physical but also emotional needs “I would like more time with them. I feel sometimes when you go in and all your levels are somewhat ok they don’t really entertain you at times” (#023), “I feel I’m rushed in and out of my appointment.. more time is needed sometimes” (#024).

### Integrating psychological care into IBD care pathways

The third theme reflected participants’ strong preference for the integration of psychological care into routine IBD management (n = 47, 31.1%) and focused on how such care should be structured. This theme contained four subthemes, “routine screening” (n = 22, 14.6%), “referral pathways” (n = 5, 3.3%), “holistic approach to care” (n = 3, 2%) and “early and sustained psychological care” (n = 21, 31.9%). Participants called for routine screening “mental health assessment” (#012), with some explicitly recommending “psychiatric assessment” (#059). Others highlighted that even being asked directly about how their IBD affected their mental health would represent a meaningful and values step in their care, “asking about or discussing such emotions” (#031). Establishing clear referral pathways was also emphasised describing the current process as “almost impossible” (#128). Participants reported a disconnect between their physical and psychological care, “it currently feels like physical healthcare and mental healthcare are completely separate things” (#079). Many believed that the psychological impact of IBD was overlooked in routine care, with consultations focused only on physical symptoms, “healthcare professionals never ask how IBD impacts your mental health, they just focus on the physical health. I have never been asked in 34 years about how I am coping” (#028). Few participants advocated for psychological intervention at the time of diagnosis, at specific timepoints in their IBD journey, and sustained psychological care overtime. One participant described the diagnosis period as “traumatic” (#025), and many called for “psychological support on diagnosis” (#064), “if any major trauma occurred” (#032), or “prior to any change in medication or surgery” (#122). The need for sustained psychological care across their IBD journey was another reoccurring preference. Some participants stressed that psychological support should be available “periodically” (#085).

### IBD specific expertise in psychological support

The fourth theme portrayed participants’ preference for high quality psychological support delivered by professionals with expertise in IBD (n = 22, 14.6%) and contained two subthemes, “specialist knowledge of IBD and mental health” (n = 8, 5.3%) and “embedded roles within IBD care” (n = 15, 9.9%). Many described frustrations when counsellors or therapists lacked an understanding of IBD, leading to repeated explanations of symptoms and treatment burdens, “the last thing a patient wants to do is have to explain themselves and their autoimmune disease to yet another person. It is hard enough to talk to the team who know most about the disease” (#029), resulting in a loss of trust “when I have to explain I lose any trust that they can help you” (#149). Others felt that there was no point in engaging with psychological services if available as “most people don’t understand what colitis is and think you have IBS or something like that. It’s totally different” (#090), “nobody understands what perianal Crohn’s is except the IBD team. There would be no point in me talking to someone because they wouldn’t get it” (#133). Several participants advocated for embedding IBD-trained psychologists or counsellors within existing IBD services, “some form of therapist should be linked with the GI service” (#014), “psychologist or psychiatrist on the IBD team” (#076).

### Expanding peer and community-based supports

The fifth and final theme reflected participants’ desire for psychological support to extend beyond formal services to peer and community-based supports (n = 35, 23.2%) and contained three subthemes, “support groups” (n = 24, 15.9%), “information provision” (n = 6, 4%), and “funding for patient organisations” (n = 13, 8.6%). Participants repeatedly called for “more support groups around the country” (#038) and the delivery format to be both online and in-person. Others emphasised the importance of information provision in the form of “postal information”, “hospital organised information nights” (#041), “leaflets” (#116) or “apps for phones” (#114). Preferred information topics included “education on services available” (#042), “where to get help if they needed it” (#072), and “information about therapy” (#086). While another participant called for “more than a website” for information and “additional resources for family members, especially for children, to help them understand better what I’m going through” (#027). Participants consistently called for “increased funding” (#054) for patient organisations such as CCI.

## Discussion

This study examined the help-seeking preferences and behaviours of individuals living with IBD in relation to negative emotions.

Participants demonstrated a moderately high intention to seek help from a healthcare professional within six months of experiencing negative emotions. Despite this, a substantial proportion (59.8%) reported experiencing negative emotions without actually seeking help, measured using past experiences of seeking help. This is consistent with patterns observed in both the IBD-specific and broader literature. For example, 50% of the total participants (n = 731) of Australians living with IBD reported emotional distress but only 15.2% were currently under the care of a mental health practitioner [20]. This discrepancy reflects a well-established intention-behaviour gap, in which individuals express a willingness to engage in a behaviour but fail to translate this into action. However, it is important to acknowledge that the present study did not longitudinally assess whether the reported intentions actually resulted in help-seeking behaviour. Nevertheless, it is thought that generally intentions are translated into action only 50% of the time [55]. Similar findings have been documented in the research regarding help-seeking for mental health, however, these international findings are interpreted as illustrative rather than directly comparable to the Irish healthcare context. A narrative review of 19 studies undertaken in New South Wales, Queensland and the Australian Capital Territory explored the factors that affect help-seeking amongst 14–24 year olds for mental health difficulties [56]. The relationship between intention and behaviour was varying but modest. Yet, Nagai [57] identified that help-seeking intentions were strongly and positively associated with actual help-seeking behaviour for social-psychological problems among university students (r = 0.53). Intentions are more likely to result in action when behaviours are within ones’ ability to perform [25]. However, the findings of this study suggest that participants experienced various perceived barriers which likely impacted their ability to act on their intentions to seek help.

Participants reported positive attitudes towards seeking help from a healthcare professionals but low levels of social pressure to do so, with the majority feeling in control of taking action. Stronger intentions were observed among those who all three constructs being positively aligned. The variance in behavioural intention explained by the model (55.5%) was relatively high compared to findings from meta-analytic reviews of the application of the TPB, which reported average variances between 39% [58] and 44.3% [59]. This higher explained variance may reflect the application of the principle of compatibility [60] to the development of the questionnaire, ensuring that all measures of attitude, subjective norms, perceived behavioural control, and intention referred to the same level of specificity. The target behaviour, seeking help for negative emotions related to IBD from a healthcare professional within six months of experiencing them, was defined in terms of target, action, context and time [36].

The findings demonstrate that attitudes and subjective norms were identified as significant predictors of behavioural intention, the latter had the strongest influence. In the meta-analytic review conducted by McEachan et al. [59], attitude, followed by perceived behaviour control and lastly subjective norms were identified as the strongest predictors. This finding was echoed by a scoping review that mapped the available literature of the TPB applied to help-seeking for mental healthcare for adults aged 18 years and older, and identified subjective norms as significant predictors of behavioural intention in 59% (n = 23) of the studies, explained by the difference in cultural norms and beliefs [25]. Similarly, Tomczyk et al. [61] investigated help-seeking behaviours among individuals in a German community sample who were experiencing untreated symptoms of depression (n = 188) and found that attitudes and subjective norms were positively related to help-seeking intention. In the setting of IBD, where the psychological challenges of living with such an illness is often under-recognised [62], social endorsement to seek help may provide those living with IBD the crucial validation that seeking help for negative emotions is both acceptable and beneficial.

Although participants generally reported a positive sense of control over seeking help from healthcare professionals, a wide range of perceived barriers to accessing support for negative emotions were also described. These included both individual and structural challenges. Although a preference for wanting to solve the problem independently, feelings of embarrassment or shame, and concerns about not being understood were quantified as common individual barriers, qualitative responses suggested that healthcare professionals did not actively ask patients regarding the psychological burden of living with IBD.

While quantitative findings identified structural barriers such as lack of awareness of available services, difficulties accessing services, and financial constraints, qualitative responses illustrated how these barriers were experienced in daily life, including difficulties in navigating referral pathways and perception of limited local services. Although direct comparisons are limited by differences in measurements scales, perceived barriers to psychotherapy have previously been reported in IBD populations. In one study (n = 187), 58% reported at least one barrier, including a perceived lack of available providers and uncertainty about access [22]. Another study (n = 162) identified lack of knowledgeable therapists (81%), cost (55%), and stigma (20%) as key barriers [63]. Collectively, these findings suggest that despite participants’ willingness and perceived ability to seek help, a complex interplay of personal, social, and systemic barriers may contribute to reduced help-seeking.

Previous help-seeking experience and a history of IBD-related surgery were the only socio-demographic or clinical characteristics statistically significantly associated with a greater intention to seek help for negative emotions related to IBD. This finding suggests that familiarity with services offering psychological support may reduce hesitancy and increase openness to seek help. In fact, only 15.4% of the sample identified a previous negative experience with a mental health service as a barrier to seeking help. A systematic review [64] investigated factors associated with healthcare utilisation for depressive and anxiety disorders in the general population, previous help-seeking experience was also associated with an increased likelihood to seek help. Additionally, a history of IBD-related surgery, possibly reflecting a more severe disease phenotype [65], was also associated with a greater intention to seek help, thus potentially increasing the recognised need for help. In comparison, the use of psychotherapy was evaluated by clinical and demographic variables [63]. Low household income (≤$30,000) and attending an IBD specialist were the only factors associated with having sought psychotherapy [63], but data on IBD-related surgery was not included in their analysis. Together, these findings indicate that familiarity with the healthcare system and more severe disease may help patients recognise the need for psychological support, and in turn, increase their willingness to engage.

The European Crohn’s and Colitis Organisation, the British Society of Gastroenterology and the American Gastroenterological Association advocate for routine psychological assessment [15, 17], integrated care pathways and MDT involvement [16]. However, participants reported barriers including cost, long waiting times, and lack of specialist knowledge, indicating that international recommendations are not consistently implemented in practice. The findings align closely with the recently published IBD Psychological Care Clinical Practice Guidelines [66], which recommended a people-centred, multidisciplinary holistic approach to care, incorporating education, promoting social connection, ensuring timely psychological input, and delivering accessible services tailored to individual needs. Findings of this study highlighted preferences for affordable, timely, and flexible access to psychological care, as well as for the delivery of such care in a range of formats to suit individual needs. Similarly, participants strong emphasis of early and sustained psychological intervention resonates with recommendations offering psychological support at times of potentially higher psychological support needs including diagnosis, during increased disease activity, hospitalisation and surgery [66]. The guidelines also highlight the importance of MDTs incorporating a psychologist (or mental health worker), a dietitian and specialist nursing staff, which corresponds with participants’ calls for increased IBD nurse capacity, and embedded psychological roles within the MDT. Notably, while the guidelines emphasise accessibility and integration of patient tailored psychological support within IBD services, they do not explicitly address cost [65]. In contrast, this study identified affordability, including calls for free psychological care as a critical patient priority, highlighting a gap between clinical recommendations and patient expressed preferences. This difference in part may be attributed to by the difference in healthcare system structures internationally. The guideline [66] was developed in Australia where a universal healthcare system, ‘Medicare’, is in operation. Ireland operates a two-tier healthcare system where only medical card holders receive fully subsidised care, and therefore, cost may be more a prohibitive factor in accessing psychological support in Ireland.

### Strength and limitations of the work

This study used non-probability voluntary response sampling via social media platforms, which may introduce self-selection bias and limit generalisability. As the sample was restricted to those living on the island of Ireland and conducted via social media, the findings may not be generalisable to IBD populations in other geographical regions or not be representative of all individuals living with IBD. Pairwise deletion was used for descriptive statistics to maximise available data [67] in this novel exploration of help-seeking behaviours for negative emotions in individuals with IBD, although this approach leads to inconsistent sample sizes across analyses [68]. The Cronbach’s alpha of 0.58 of the subjective norm scale, indicated lower internal consistency, which may have potentially reduced precision and observed associations. Furthermore, the post-hoc removal of one item from the perceived behavioural control scale to improve reliability represents a limitation. Such removal after data collection may affect construct validity and limit replicability. Although content validity was systematically assessed, several items included in the questionnaire were researcher-developed and not previously validated in other populations. Therefore, measurement error cannot be excluded and findings related to these questions should be interpreted with caution. Finally, due to the cross-sectional correlational design, it was not possible to determine whether the reported intentions translated into help-seeking behaviour later, or whether modifying the identified barriers would meaningfully increase engagement with support. Overall, the findings of this study offer meaningful insights into help-seeking for negative emotions related to IBD. These findings would be best understood in the context of the study’s limitations, as outlined.

Notwithstanding these limitations, the study has several significant strengths. To our knowledge, it is the first study to apply the TPB to help-seeking for negative emotions related to IBD, enhancing the conceptual understanding of the independent concepts that govern behavioural intention to seek help for such emotions. It is also the first study on the island of Ireland to examine preferences, intentions and behaviours in this context. However, the findings may also be applicable to international healthcare systems with a similar structure to the Irish system. Additionally, the study is theory-driven by the TPB which offers a robust conceptual framework used to guide the design and interpretation of findings. Finally, the study highlights the psychological burden experienced by those living with IBD and the notable lack of adequate support services available to them at an afforable cost.

### Recommendations for further research

The findings generate hypotheses regarding the intention-behaviour gap in psychological within the patient cohort under examination. Longitudinal research may help to examine how help-seeking intention change over time and under what conditions they translate into behaviour. Further research using explanatory sequential mixed-methods design could explore how healthcare system structures and the practices of healthcare professionals influence patients’ help-seeking behaviours. Such work would generate hypotheses regarding how factors such as service accessibility, availability, and practices of healthcare professionals may influence the translation of intention into behaviour, which could be examined in future confirmatory research.

### Implications for policy and practice

Given the cross-sectional and correlational design of this study, casual inferences cannot be drawn. Therefore, changes to practice or policy should not be based solely on the findings of this study. However, the participants reported substantial psychological burden and identified barriers to accessing formal psychological support. These findings align with international best practice guidelines which advocate for the routine assessment of the psychological impact and health-related quality of life at clinic visits [15, 17]. This study provides empirical support for the implementation of these international best practice guidelines that encourage healthcare professionals to ask patients regards the impact of their IBD on their psychological wellbeing, facilitate open discussions, and improve awareness of available mental health resources. While the effectiveness of specific implementation strategies cannot be determined from the cross-sectional, correlational study design, the identified barriers and participants’ intention patterns highlight the relevance of viewing psychological wellbeing as an important component of IBD care.

## Conclusion

The aim of this study was to explore the help-seeking preferences and behaviours of those living with IBD for negative emotions related to their disease. Specifically, it assessed participants’ behavioural intentions to seek help and determined the predictive utility of the TPB in explaining such intentions. Additionally, the study described barriers and examined factors associated with accessing formal psychological support, as well as preferences for such support. The findings demonstrated that although participants reported moderately high intentions to seek help, a substantial amount had failed to do so. The strongest predictors of intention were subjective norms and attitudes, highlighting the influence of perceived social expectations and personal beliefs. Several key barriers were also identified such as a lack of service awareness, a desire for wanting to solve the problem independently, and feelings of embarrassment or shame. Previous help-seeking experience and a history of IBD-related surgery were associated with stronger intentions to seek help. Participants call for the integration of psychological care into routine IBD care and made free or affordable to mitigate cost as a barrier to access. Although casual relationships cannot be inferred from the cross-sectional, correlational study design, the findings provide behavioural insight into factors associated with seeking help for negative emotions related to IBD and add to the growing body of literature emphasising the relevance of viewing psychological wellbeing as an important component of IBD care.

## Supporting information

S1 File Behavioural Intention Items. Tables 3 and 4, Figures 1 and 2.
(DOCX)

S2 File Formal and Informal Sources of Support. Table 5.
(DOCX)

S3 File Correlations Between Behavioural Intention and the Constructs of the TPB. Figures 3–5.
(DOCX)

S4 File Attitude Items. Tables 6 and 7, Figures 6 and 7.
(DOCX)

S5 File Subjective Norm Items. Tables 8 and 9, Figures 8 and 9.
(DOCX)

S6 File Perceived Behavioural Control Items. Tables 10 and 11, Figures 10 and 11.
(DOCX)

S7 File Barriers to Accessing Help. Table 12.
(DOCX)

S8 File Standard and Hierarchical Multiple Regression Plots. (DOCX)
