# Supplementary material for: Psychological help-seeking behaviours amongst those living with Inflammatory Bowel Disease; A cross-sectional, descriptive, correlational study
Source: PLoS One. 2026 Apr 10;21(4):e0346243. doi: 10.1371/journal.pone.0346243 (PMC13068262; doi:10.1371/journal.pone.0346243)
Supplement: S1 File — Tables 3 and 4, Figures 1 and 2. (DOCX) [file pone.0346243.s001.docx]

**Supplementary File 1. Behavioural Intention Items.**

**Figure 1. Histogram of Mean Behavioural Intention.**

**
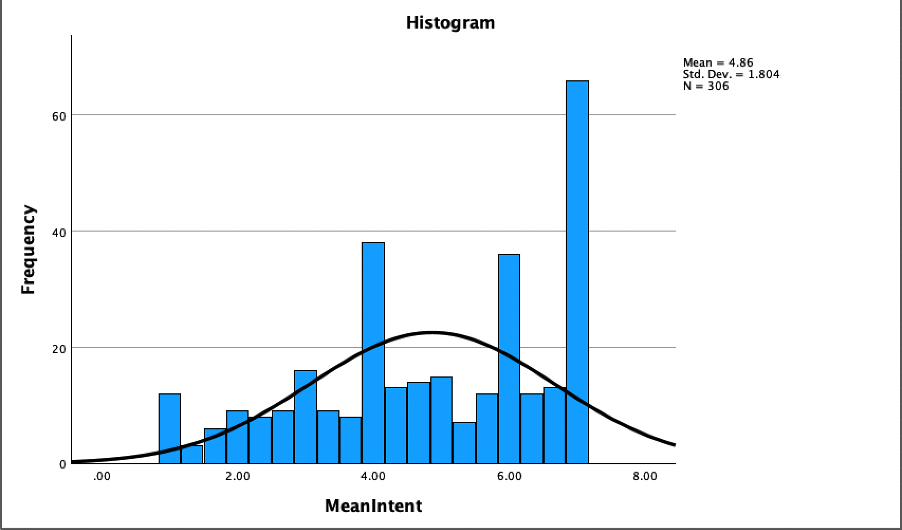
**

Distribution of mean behavioural intention scores for seeking psychological support from a healthcare professional for negative emotions related to their IBD.

**Figure 2. Q-Q Plot of Mean Behavioural Intention.**

**
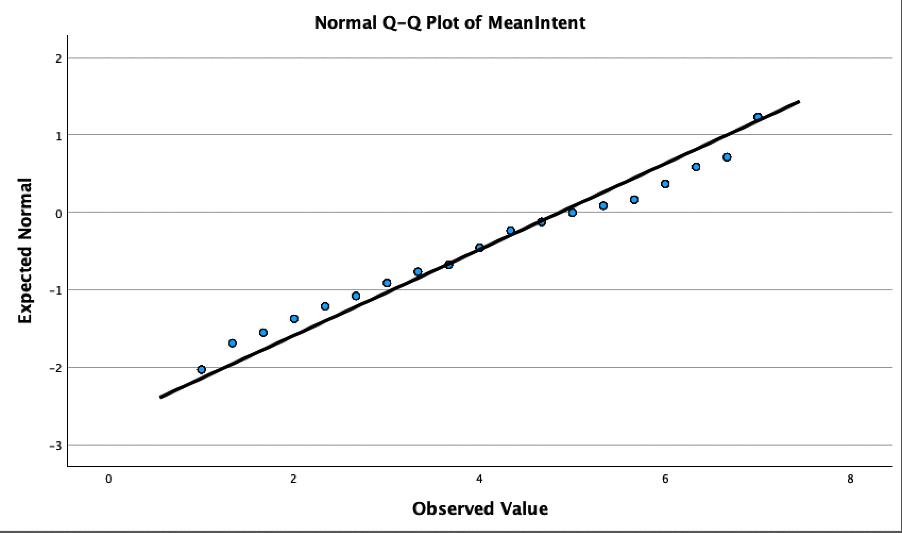
**

Q-Q plot of mean behavioural intention scores for seeking psychological support from a healthcare professional for negative emotions related to IBD.

**Table 3. Behavioural Intention Item-Level Responses.**

| **Item^ab^** | **Percentage Response** | | | | | | |
| --- | --- | --- | --- | --- | --- | --- | --- |
|  | **Strongly disagree**  **1** | **2** | **3** | **4** | **5** | **6** | **Strongly agree**  **7** |
| S5, Q1  I expect to seek help from a healthcare professional for negative emotions related to my Inflammatory Bowel Disease | 8.8%  (n=27) | 5.9%  (n=18) | 9.8%  (n=30) | 19.9%  (n=61) | 9.2%  (28) | 16.7%  (n=51) | 29.7%  (n=91) |
|  | **Strongly disagree**  **1** | **2** | **3** | **4** | **5** | **6** | **Strongly agree**  **7** |
| S5, Q2  I intend to seek help from a healthcare professional for negative emotions related to my Inflammatory Bowel Disease | 11.8%  (n=36) | 5.6%  (n=17) | 9.2%  (n=28) | 18.3%  (n=56) | 9.5%  (n=29) | 17.0%  (n=52) | 28.8%  (n=88) |
|  | **Strongly disagree**  **1** | **2** | **3** | **4** | **5** | **6** | **Strongly agree**  **7** |
| S5, Q13  I want to seek help from a healthcare professional for negative emotions related to my Inflammatory Bowel Disease | 8.8%  (n=27) | 4.9%  (n=15) | 3.3%  (n=10) | 21.2%  (n=65) | 13.1%  (n=40) | 18.0%  (n=55) | 30.7%  (n=94) |

^a^ (n=306, missing data n=70)

^b^ S=Section, Q= Question

**Table 4. Descriptive Statistics for Behavioural Intention Item-Level Responses.**

| **Item^ab^** | **Mean** | **SD^b^** |
| --- | --- | --- |
| S5, Q1 | 4.84 | 1.968 |
| S5, Q2 | 4.74 | 2.0949 |
| S5, Q13 | 5.02 | 1.902 |
| Total | 4.8649 | 1.80384 |

^a^ (n=306, missing data n=70)

^b^ S=Section, Q= Question, SD= Standard Deviation
