# Supplementary material for: Psychological help-seeking behaviours amongst those living with Inflammatory Bowel Disease; A cross-sectional, descriptive, correlational study
Source: PLoS One. 2026 Apr 10;21(4):e0346243. doi: 10.1371/journal.pone.0346243 (PMC13068262; doi:10.1371/journal.pone.0346243)
Supplement: S2 File — Table 5. (DOCX) [file pone.0346243.s002.docx]

**Supplementary File 2. Formal and Informal Sources of Support.**

**Table 5. Formal and Informal Sources of Support.**

| **Formal Sources^a^** | **% (n)** |
| --- | --- |
| GP | 21 (79) |
| IBD nurse | 20.5 (77) |
| Counsellor | 10.9 (41) |
| Psychologist | 4.8 (18) |
| Other^b^  Other healthcare professionals  Charitable organisation  Complimentary therapist  Online sources of support | 3.2 (12)  (4)  (3)  (1)  (1) |
| Psychotherapist | 2.4 (9) |
| Psychiatrist | 1.3 (5) |
| Mental health social worker | 0.5 (2) |
| Behavioural therapist | 0.3 (1) |
| **Informal Sources^a^** | **% (n)** |
| Partner/significant other | 44.9 (169) |
| Friend | 34.3 (129) |
| Another person living with IBD | 30.9 (116) |
| Parent | 23.4 (88) |
| Sibling | 20.5 (77) |
| Another relative/family member | 9.8 (37) |
| Other^b^  Online sources of support  Complimentary therapists  Work colleagues  Healthcare professionals  Charitable organisations  Another person living with IBD | 4.8 (18)  (4)  (4)  (2)  (2)  (1)  (1) |
| Religious/spiritual leader | 1.3 (5) |

^a^ Participants could select multiple sources of support.

^b^ Not all participants provided a breakdown of ‘other’ sources of support.
