# Supplementary material for: Psychological help-seeking behaviours amongst those living with Inflammatory Bowel Disease; A cross-sectional, descriptive, correlational study
Source: PLoS One. 2026 Apr 10;21(4):e0346243. doi: 10.1371/journal.pone.0346243 (PMC13068262; doi:10.1371/journal.pone.0346243)
Supplement: S3 File — Figures 3–5. (DOCX) [file pone.0346243.s003.docx]

**Supplementary File 3. Correlations Between Behavioural Intention and the Constructs of the TPB.**

**Figure 3: Scatterplot of Mean Attitude by Mean Intention**

**
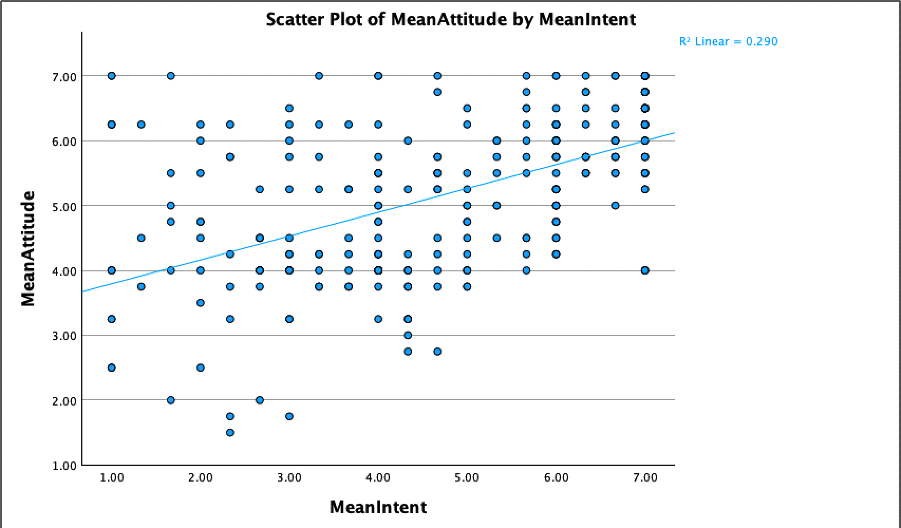
**

Scatterplot showing the association between mean attitudes and help-seeking intentions.

Figure 4: Scatterplot of Mean Subjective Norms by Mean Intention


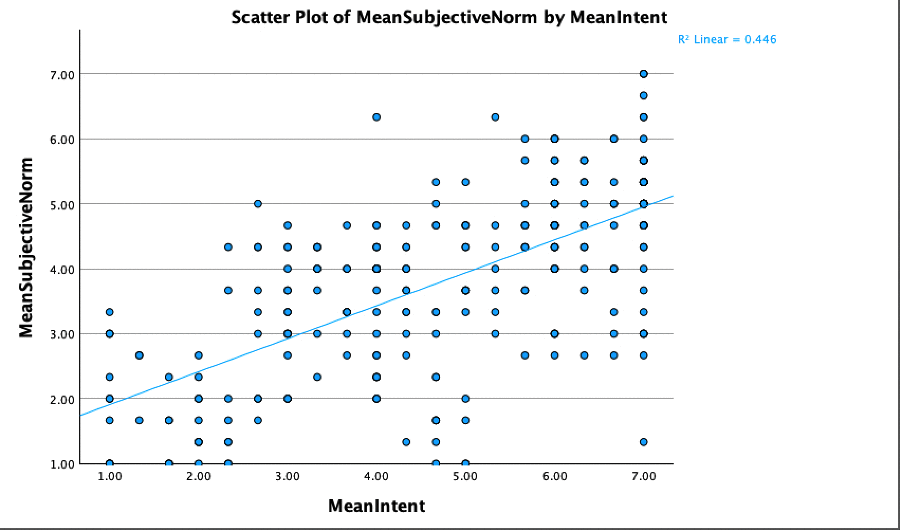


Scatterplot showing the association between mean subjective norms and help-seeking intentions.

Figure 5: Scatterplot of Mean Perceived Behavioural Control by Mean Intention.


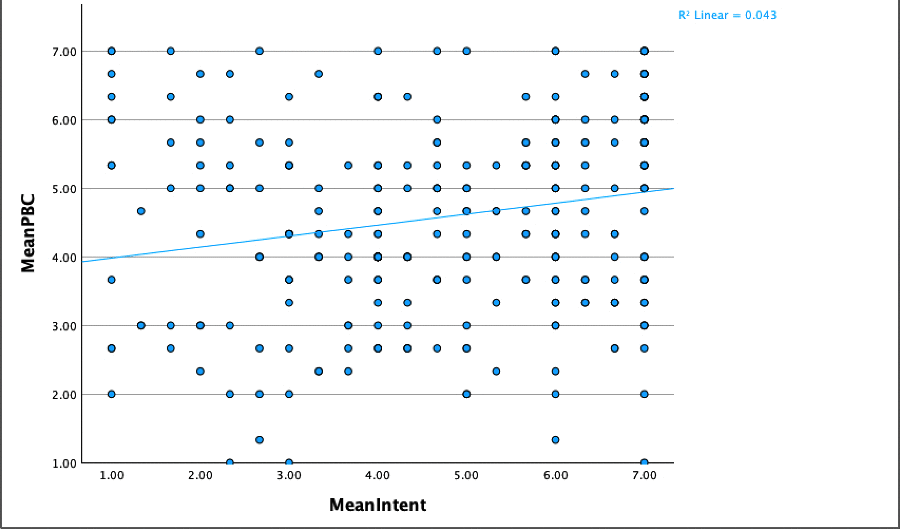


Scatterplot showing the association between mean perceived behavioural control and help-seeking intentions.
