# Supplementary material for: Psychological help-seeking behaviours amongst those living with Inflammatory Bowel Disease; A cross-sectional, descriptive, correlational study
Source: PLoS One. 2026 Apr 10;21(4):e0346243. doi: 10.1371/journal.pone.0346243 (PMC13068262; doi:10.1371/journal.pone.0346243)
Supplement: S4 File — Tables 6 and 7, Figures 6 and 7. (DOCX) [file pone.0346243.s004.docx]

**Supplementary File 4. Attitude Items.**

**Figure 6. Histogram of Mean Attitude.**

**
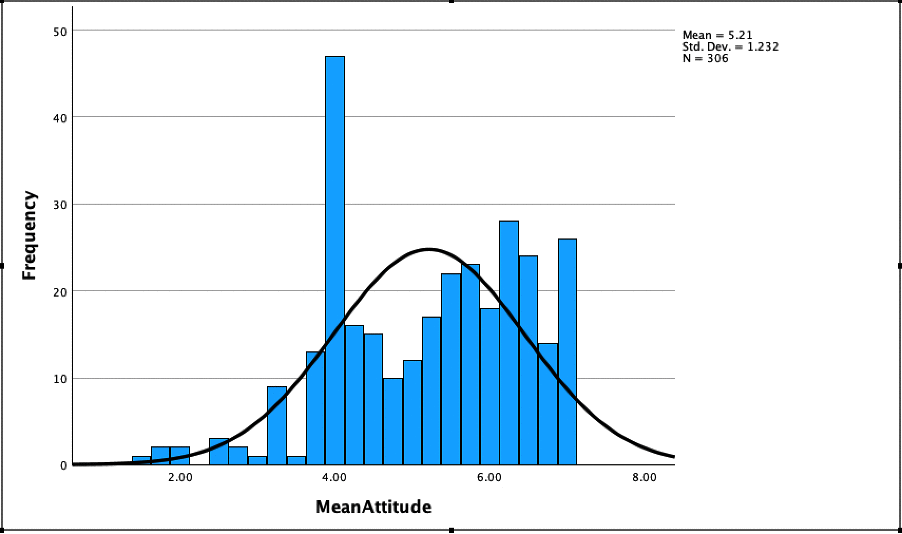
**

Distribution of mean attitude scores for seeking psychological support from a healthcare professional for negative emotions related to IBD.

**Figure 7. Q-Q Plot of Mean Attitude.**

**
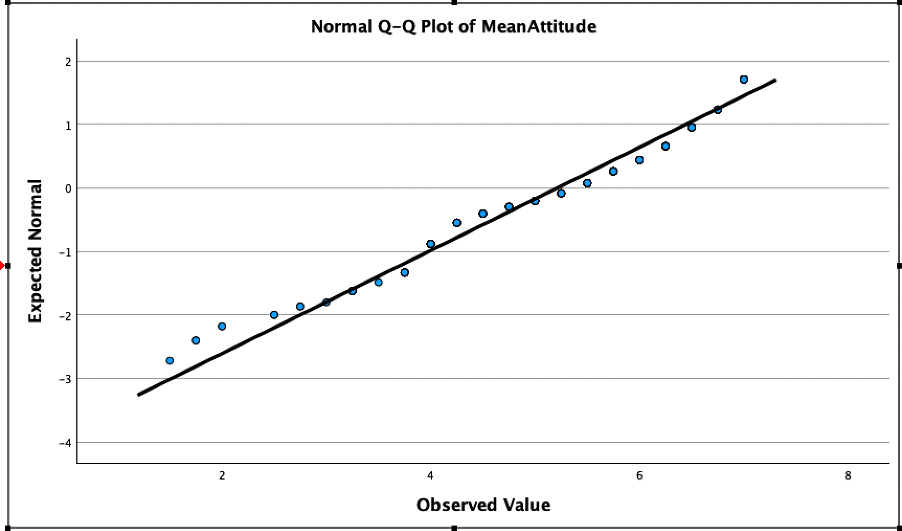
**

Q-Q plot of mean attitude scores for seeking psychological support for negative emotions related to IBD.

**Table 6: Attitude Item-Level Responses.**

| **Item^abc^** | **Percentage Responses** | | | | | | |
| --- | --- | --- | --- | --- | --- | --- | --- |
| S5, Q3 | Harmful  1 | 2 | 3 | 4 | 5 | 6 | Beneficial  7 |
|  | 1.6% (n=5) | 1.3% (n=4) | 1.0% (n=3) | 19.6% (n=60) | 10.5% (n=32) | 18.0% (n=55) | 48% (n=147) |
| S5, Q4 | Bad  1 | 2 | 3 | 4 | 5 | 6 | Good  7 |
|  | 2.9%  (n=9) | 3.6%  (n=11) | 3.3%  (n=10) | 21.6%  (n=66) | 9.8%  (n=30) | 13.7% (n=42) | 45.1%  (n=138) |
| S5, Q5 | Unpleasant (for me)  1 | 2 | 3 | 4 | 5 | 6 | Pleasant (for me)  7 |
|  | 9.8%  (n=30) | 9.2%  (n=28) | 9.2%  (n=28) | 32.7%  (n=100) | 17.3%  (n=53) | 10.5%  (n=32) | 11.4%  (n=35) |
| S5, Q6 | Worthless  1 | 2 | 3 | 4 | 5 | 6 | Useful  7 |
|  | 4.2%  (n=13) | 2.0%  (n=6) | 5.2%  (n=16) | 21.2%  (n=65) | 13.1%  (n=40) | 19.3%  (n=59) | 35%  (n=107) |

^a^ All items were preceded by the stem: *“Seeking help from a healthcare professional for negative emotions related to my IBD is …”*

^b^ (n=306, missing data n=70).

^c^ S=Section, Q= Question.

**Table 7: Descriptive Statistics for Attitude Item-Level Responses.**

| **Item^abc^** | **Mean** | **SD** |
| --- | --- | --- |
| S5, Q3 | 5.82 | 1.432 |
| S5, Q4 | 5.53 | 1.675 |
| S5, Q5 | 4.16 | 1.716 |
| S5, Q6 | 5.35 | 1.661 |
| Total | 5.21 | 1.232 |

^a^ All items were preceded by the stem: *“Seeking help from a healthcare professional for negative emotions related to my IBD is …”*

^b^ (n=306, missing data n=70).

c S=Section, Q= Question, SD= Standard Deviation.
