# Supplementary material for: Psychological help-seeking behaviours amongst those living with Inflammatory Bowel Disease; A cross-sectional, descriptive, correlational study
Source: PLoS One. 2026 Apr 10;21(4):e0346243. doi: 10.1371/journal.pone.0346243 (PMC13068262; doi:10.1371/journal.pone.0346243)
Supplement: S5 File — Tables 8 and 9, Figures 8 and 9. (DOCX) [file pone.0346243.s005.docx]

**Supplementary File 5. Subjective Norm Items.**

**Figure 8. Histogram of Mean Subjective Norm.**

**
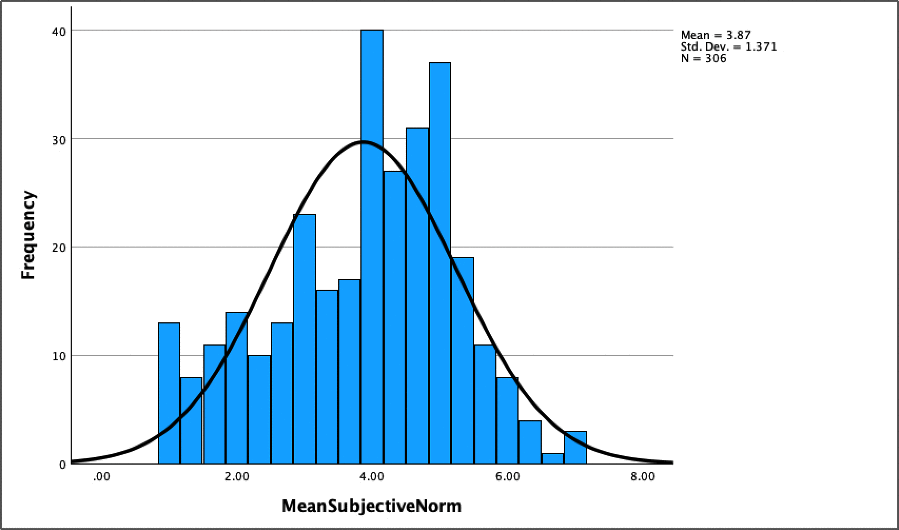
**

Distribution of mean subjective norm scores for seeking psychological support from a healthcare professional for negative emotions related to IBD.

**Figure 9. Q-Q Plot of Mean Subjective Norm.**

**
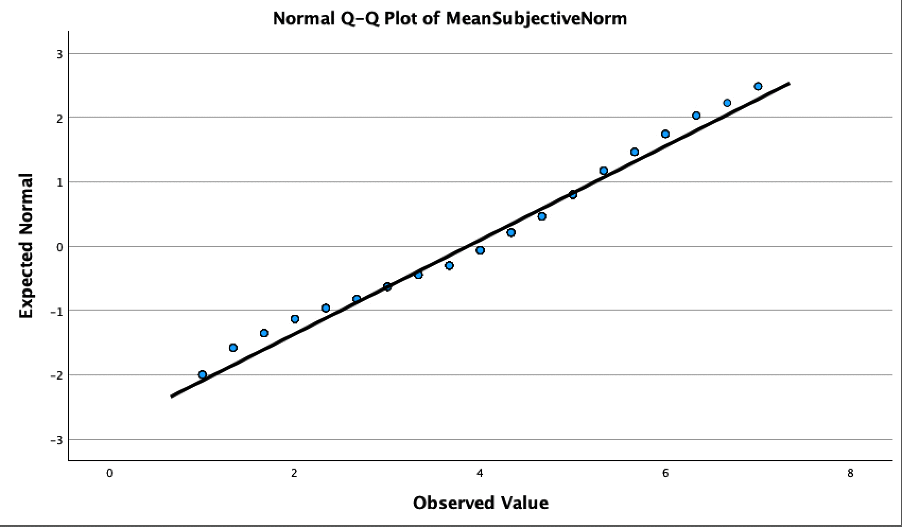
**

Q-Q plot of mean subjective norm scores for seeking psychological support for negative emotions related to IBD.

**Table 8: Subjective Norms Item-Level Responses.**

| **Item^ab^** | **Percentage Responses** | | | | | | |
| --- | --- | --- | --- | --- | --- | --- | --- |
|  | **Strongly disagree**  **1** | **2** | **3** | **4** | **5** | **6** | **Strongly agree**  **7** |
| S5, Q8  Most people who are important to me think that I should seek help from a healthcare professional for negative emotions related to my Inflammatory Bowel Disease | 12.7%  (n=39) | 6.5%  (n=20) | 7.8%  (n=24) | 20.6%  (n=63) | 13.1%  (n=40) | 20.3%  (n=62) | 19.0%  (n=58) |
| S5, Q10  It is expected of me that I seek help from a healthcare professional for negative emotions related to my Inflammatory Bowel Disease | 15.4%  (n=47) | 6.2%  (n=19) | 8.5%  (n=26) | 23.5%  (n=72) | 19.0%  (n=58) | 15.0%  (n=46) | 12.4%  (n=38) |
| S5, Q14  I feel under social pressure to seek help from a healthcare professional for negative emotions related to my Inflammatory Bowel Disease | 30.7%  (n=94) | 14.1%  (n=43) | 17.0%  (n=52) | 20.9%  (n=64) | 11.4%  (n=35) | 1.6%  (n=5) | 4.2%  (n=13) |

^a^ (n=306, missing data n=70).

^b^ S=Section, Q= Question.

**Table 9: Descriptive Statistics for Subjective Norms Item-Level Responses.**

| **Item^ab^** | **Mean** | **SD** |
| --- | --- | --- |
| S5, Q8 | 4.51 | 1.971 |
| S5, Q10 | 4.19 | 1.897 |
| S5, Q14 | 2.90 | 1.692 |
| Total | 3.87 | 1.37 |

^a^ (n=306, missing data n=70).

^b^ S=Section, Q= Question, SD= Standard Deviation.
