# Supplementary material for: Psychological help-seeking behaviours amongst those living with Inflammatory Bowel Disease; A cross-sectional, descriptive, correlational study
Source: PLoS One. 2026 Apr 10;21(4):e0346243. doi: 10.1371/journal.pone.0346243 (PMC13068262; doi:10.1371/journal.pone.0346243)
Supplement: S6 File — Tables 10 and 11, Figures 10 and 11. (DOCX) [file pone.0346243.s006.docx]

**Supplementary File 6. Perceived Behavioural Control Items.**

**Figure 10. Histogram of Mean Perceived Behavioural Control.**

**
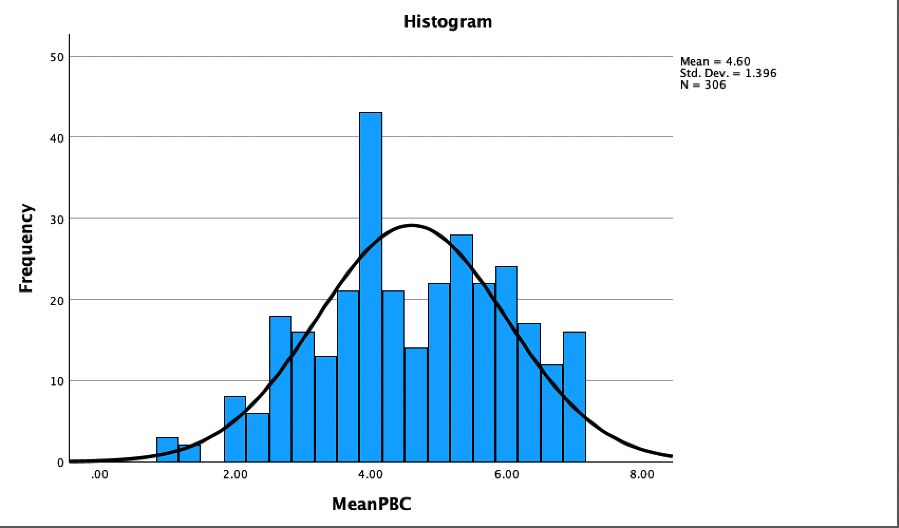
**

Distribution of mean perceived behavioural control scores for seeking psychological support from a healthcare professional for negative emotions related to IBD.

**Figure 11. Q-Q Plot of Mean Perceived Behavioural Control.**

**
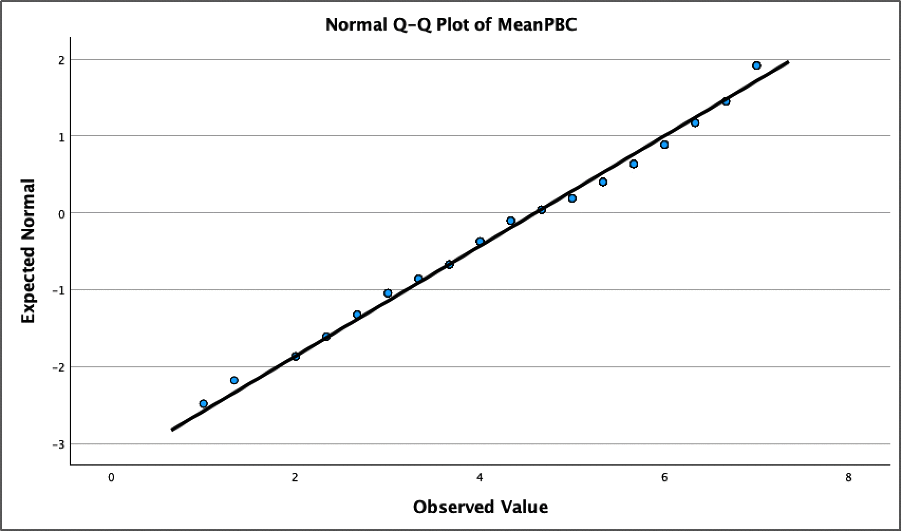
**

Q-Q plot of mean perceived behavioural control scores for seeking psychological support from a healthcare professional for negative emotions related to IBD.

**Table 10: Perceived Behavioural Control Item-Level Responses.**

| **Item^ab^** | **Percentage Responses** | | | | | | |
| --- | --- | --- | --- | --- | --- | --- | --- |
|  | Strongly disagree  1 | 2 | 3 | 4 | 5 | 6 | Strongly agree  7 |
| S5, Q9  I am confident that I could seek help from a healthcare professional for negative emotions related to my Inflammatory Bowel Disease if I wanted to | 4.9%  (n=15) | 5.2%  (n=16) | 9.2%  (n=28) | 15.7%  (n=48) | 13.7%  (n=42) | 25.5%  (n=78) | 25.8%  (n=79) |
|  | Difficult  1 | 2 | 3 | 4 | 5 | 6 | Easy  7 |
| S5, Q11  For me to seek help from a healthcare professional for negative emotions related to my Inflammatory Bowel Disease is | 15.7%  (n=48) | 10.5%  (n=32) | 15.0%  (n=46) | 23.2%  (n=71) | 17.6%  (n=54) | 11.4%  (n=35) | 6.5%  (n=20) |
|  | Strongly disagree  1 | 2 | 3 | 4 | 5 | 6 | Strongly agree  7 |
| S5, Q12  The decision to seek help from a healthcare professional for negative emotions related to my Inflammatory Bowel Disease is beyond my control | 4.6%  (n=14) | 5.6%  (n=17) | 7.8%  (n=24) | 23.5%  (n=72) | 14.1%  (n=43) | 19.3%  (n=59) | 25.2%  (n=77) |

^a^ (n=306, missing data n=70).

^b^ S=Section, Q= Question.

**Table 11: Descriptive Statistics for Perceived Behavioural Control Item-Level Responses.**

| **Item** | **Mean** | **SD** |
| --- | --- | --- |
| S5, Q9 | 5.08 | 1.752 |
| S5, Q11 | 3.77 | 1.778 |
| S5, Q12 | 4.95 | 1.729 |
| Total | 4.60 | 1.396 |

^a^ (n=306, missing data n=70).

^b^ S=Section, Q= Question, SD= Standard Deviation.
