# Supplementary material for: Psychological help-seeking behaviours amongst those living with Inflammatory Bowel Disease; A cross-sectional, descriptive, correlational study
Source: PLoS One. 2026 Apr 10;21(4):e0346243. doi: 10.1371/journal.pone.0346243 (PMC13068262; doi:10.1371/journal.pone.0346243)
Supplement: S7 File — Table 12. (DOCX) [file pone.0346243.s007.docx]

**Supplementary File 7. Barriers to Accessing Help.**

**Table 12. Barriers to Accessing Help.**

| **Lack of knowledge of available services^a^** | **% (n)** |
| --- | --- |
| Strongly disagree | 4.5 (15) |
| Disagree | 7.8 (26) |
| Neutral | 19.8 (66) |
| Agree | 39.2 (131) |
| Strongly agree | 28.7 (96) |
| **Being unable to access services^b^** | **% (n)** |
| Strongly disagree | 4.8 (16) |
| Disagree | 9.6 (32) |
| Neutral | 28 (93) |
| Agree | 29.2 (97) |
| Strongly agree | 28.3 (94) |
| **Being unable to attend appointments^b^** | **% (n)** |
| Strongly disagree | 10.2 (34) |
| Disagree | 18.7 (62) |
| Neutral | 23.5 (78) |
| Agree | 28.9 (96) |
| Strongly agree | 18.7 (62) |
| **Feeling embarrassed or ashamed^b^** | **% (n)** |
| Strongly disagree | 8.4 (28) |
| Disagree | 9.3 (31) |
| Neutral | 22 (73) |
| Agree | 33.1 (110) |
| Strongly agree | 27.1 (90) |
| **Being unable to afford the associated costs^c^** | **% (n)** |
| Strongly disagree | 7.5 (25) |
| Disagree | 13.5 (45) |
| Neutral | 22.8 (76) |
| Agree | 22.5 (75) |
| Strongly agree | 33.6 (112) |
| **Wanting to solve the problem by myself^c^** | **% (n)** |
| Strongly disagree | 4.2 (14) |
| Disagree | 9.6 (32) |
| Neutral | 22.2 (74) |
| Agree | 41.4 (138) |
| Strongly agree | 22.5 (75) |
| **Previous bad experience of attending mental health services^d^** | **% (n)** |
| Strongly disagree | 37.5 (124) |
| Disagree | 18.7 (62) |
| Neutral | 28.4 (94) |
| Agree | 10.3 (34) |
| Strongly agree | 5.1 (17) |
| **Thinking that the treatment offered wouldn’t help^c^** | **% (n)** |
| Strongly disagree | 8.1 (27) |
| Disagree | 11.4 (38) |
| Neutral | 37.2 (124) |
| Agree | 36 (120) |
| Strongly agree | 7.2 (24) |
| **Feeling that I wouldn’t be understood^a^** | **% (n)** |
| Strongly disagree | 7.5 (25) |
| Disagree | 12.0 (40) |
| Neutral | 25.7 (86) |
| Agree | 33.5 (112) |
| Strongly agree | 21.3 (71) |
| **Believing that others needed more help than me^b^** | **% (n)** |
| Strongly disagree | 8.4 (28) |
| Disagree | 10.2 (34) |
| Neutral | 37.0 (123) |
| Agree | 27.7 (92) |
| Strongly agree | 16.6 (55) |

Barriers to accessing help for negative emotions related to IBD.

^a^ (n=334, missing data n=42)

^b^ (n=332, missing data n=44)

^c^(n=333, missing data n=43)

^d^ (n=331, missing data n=45)
