# Supplementary material for: Psychological help-seeking behaviours amongst those living with Inflammatory Bowel Disease; A cross-sectional, descriptive, correlational study
Source: PLoS One. 2026 Apr 10;21(4):e0346243. doi: 10.1371/journal.pone.0346243 (PMC13068262; doi:10.1371/journal.pone.0346243)
Supplement: S8 File — (DOCX) [file pone.0346243.s008.docx]

**Supplementary File 8. Standard and Hierarchical Multiple Regression Plots.**

Fig 12. Standard Multiple Regression Plot.





Scatterplot showing the distribution of standardised residuals against standardised predicted values for the standard multiple regression model examining the influence of attitude, subjective norms, and perceived behavioural control on behavioural intention. The plot indicates that the assumption of homoscedasticity was met.

Fig 13. Hierarchical Multiple Regression Residual Plot (Final Model).





Scatterplot displaying the standardised residuals plotted against the standardised predicted values for the final model (Model 4) of the hierarchical multiple regression. The plot indicates that the assumption of homoscedasticity was met.
